# An Observational Study Investigating the Need for Decompressive Hemicraniectomy after Thrombectomy in Acute Ischemic Stroke of the Middle Cerebral Artery Territory

**DOI:** 10.7759/cureus.9665

**Published:** 2020-08-11

**Authors:** Arsalaan Salehani, Borna E Tabibian, D M Self, Bonita Agee, Gustavo Chagoya, William Stetler, Winfield S Fisher

**Affiliations:** 1 Neurological Surgery, University of Alabama at Birmingham, Birmingham, USA

**Keywords:** stroke, hemicraniectomy, thrombectomy, tici

## Abstract

Objective: The frequency incidence of decompressive hemicraniectomy following intra-arterial thrombectomy (IAT) in acute ischemic stroke (AIS) involving the middle cerebral artery (MCA) territory was assessed as a surrogate for morbidity.

Methods: A single-institution retrospective chart review was conducted involving 209 consecutive patients between September 2014 and May 2017 with infarctions affecting the MCA territory and who subsequently underwent IAT. The outcomes of interest included the frequency of hemicraniectomy following IAT and the effects of intravenous tissue plasminogen activator (IV tPA) use and primary occlusion site on the Thrombolysis in Cerebral Infarction (TICI) score.

Results: Thirty-one patients were excluded for infarctions not involving the MCA territory. A total of 178 patients were included in the study. Sixty-eight patients (38.6%) had infarctions of less than one-third of the MCA territory, 50 (28.4%) had infarctions between one-third and two-thirds, and 58 (33%) had infarctions involving greater than two-thirds with 54.3% suffering infarctions of the left side. Only four patients (2.2%) required a hemicraniectomy with no statistically significant association found between TICI score and hemicraniectomy (p=0.41) or between administration of IV tPA and hemicraniectomy (p=0.36). The primary occlusion site was found to influence TICI score (p=0.045).

Conclusion: A very small number of patients required hemicraniectomy after IAT as compared to previously published rates in the literature. However, several factors may prevent the patient from being an appropriate hemicraniectomy candidate in the first place and the small number of these patients in this study limits statistical analysis. The variables that determine a patient’s candidacy for decompressive hemicraniectomy remains multi-factorial.

## Introduction

Acute ischemic stroke (AIS) is one of the leading causes of morbidity in the United States [1]. However, recent technical improvements in thrombectomy devices have led to higher recanalization rates [2-5]. Increased rates of recanalization are associated with higher rates of patient survival, including improved functional outcomes [6,7].

The Thrombolysis in Cerebral Infarction (TICI) grading system was designed to be the standard for reporting the results of intra-arterial thrombectomy (IAT) following AIS [8]. Recent studies have examined the TICI grading system to determine what should define “successful” reperfusion [9-11]. The desired outcome of treatment is complete reperfusion; however, the differences in functional outcomes and clinical relevance based on reperfusion scores is still debated [9,10,12].

Decompressive hemicraniectomy is a procedure in which a large bone flap is removed to allow the brain to expand extracranially, alleviating increased intracranial pressure, and has been shown to be a life-saving procedure in the setting of complete middle cerebral artery (MCA) infarction [13-16]. Following IAT, decompressive hemicraniectomy may be used as emergent management for malignant cerebral edema and/or hemorrhage associated with the infarction [17,18]. It has been suggested that innovations in endovascular techniques may now be decreasing the incidence of hemicraniectomy [19-22].

The primary outcome measures of this study was to determine the incidence of patients undergoing decompressive hemicraniectomy following IAT and whether TICI score affects the rates of hemicraniectomy after thrombectomy as a surrogate for stroke associated morbidity. We hypothesize that even without complete recanalization, patients who obtain any significant reperfusion are at decreased risk of requiring decompressive hemicraniectomy. Additional outcomes of interest included correlation of hemicraniectomy with use of intravenous tissue plasminogen activator (IV tPA) and with site of vascular occlusion. Overall, mortality was also investigated as an outcome of interest correlated with TICI score, IV tPA use, and site of vascular occlusion. 

## Materials and methods

Study population

Institutional review board approval was obtained for this study (IRB#x170424003). A retrospective chart review was completed for all consecutive patients with infarction involving the MCA vascular territory who underwent IAT between September 2014 and May 2017. The patients were then divided based on TICI score achieved following IAT. Grade 0 was considered no perfusion, grade 1 was considered penetration of thrombus with minimal perfusion, grade 2a was considered less than 2/3 of the entire vascular territory visualized, grade 2b was considered greater than 2/3 of the entire vascular territory visualized, and grade 3 was considered complete perfusion of the vascular territory. Data on age, sex, race, mortality, and comorbidities (e.g. tobacco use, atrial fibrillation, diabetes, hypertension, hyperlipidemia, and coronary artery disease) were also collected. The primary outcome was the need for decompressive hemicraniectomy. The secondary outcome of patient mortality was investigated with TICI score, IV tPA use, and site of occlusion considered as possible associated variables.

Image assessment

Stroke protocol MRI was used to assess the fraction of the MCA vascular territory involved, with diffusion-weighted imaging used as the primary sequence of interest. Imaging was reviewed by a neurosurgery resident and a senior interventional neuroradialogist at the University of Alabama at Birmingham Department of Neurosurgery. The fraction of the MCA vascular territory involved was divided into three categories: less than one-third, one-third to two-thirds, and greater than two-thirds. The TICI score was self-reported by the neuro-interventionalist performing the procedure, as documented in the patient’s chart. 

Statistical analysis

Frequency distributions of demographics, clinical characteristics, and comorbidities were obtained. Chi-square tests and Fisher’s exact tests, where appropriate, were used to determine the statistical significance of any differences according to TICI scores, IV tPA administration, and vital status when compared with both the need for hemicraniectomy and overall mortality. The variable representing fraction of MCA territory infarction was categorized as less than one-third, between one-third and two-thirds, and greater than two-thirds MCA territory infarction. Logistic regression analysis was used to explore predictors of mortality using selected variables found to be statistically significant in bivariate analysis. Statistical significance was defined as p < 0.05. All statistical analysis were performed using SAS version 9.4 (SAS Institute Inc., Cary, NC).

## Results

A total of 178 patients were included in the study. 54.5% (n=97) were male and 45.5% (n=81) were female. Atrial fibrillation (n=35), hyperlipidemia (n=51), and coronary artery disease (n=20) were found to influence mortality following endovascular treatment (p=0.005, 0.005, and 0.03 respectively) but did not influence TICI score (p=0.51, 0.64, and 0.64 respectively).

38.6% (n=68) of patients had infarctions of less than one-third of the MCA territory, 28.4% (n=50) had infarctions between one-third and two-thirds, and 33.0% (n=58) had infarctions involving greater than two-thirds of the MCA territory. The fraction of MCA territory infarcted was found to influence patient mortality (p=<0.0001). The infarct was found to involve the left hemisphere in 54.3% (n=94) of patients. There was no statistically significant difference based on the side of the infarct on mortality or TICI score (p=0.35 and 0.72, respectively) (Table 1).

**Table 1:** Baseline and selected characteristics of study patients by TICI scores TICI: Thrombolysis in Cerebral Infarction score; MCA: middle cerebral artery; IV tPA: intravenous tissue plasminogen activator.

Table 1. Baseline and selected characteristics of study patients by TICI scores								
			TICI Scores					
Variable	Total N=178		Grade 0 n=17	Grade 1 n=8	Grade 2a n=24	Grade 2b n=61	Grade 3 n=67	P-value
									Gender, n (%)								0.41
Male	97 (54.5)		12 (70.6)	3 (33.3)	14 (58.3)	34 (55.7)	32 (50.7)	
Female	81 (45.5)		5 (29.4)	6 (66.7)	10 (41.7)	27 (44.3)	33 (49.3)	
Race, n (%)								0.15
White	110 (65.5)		10 (62.5)	8 (88.9)	11 (47.8)	37 (67.3)	44 (67.7)	
Black	56 (33.3)		5 (31.2)	1 (11.1)	11 (47.8)	18 (32.7)	21 (32.3)	
Asian	2 (1.2)		1 (6.3)	0 (0.0)	1 (4.4)	0 (0.0)	0 (0.0)	
Hemicraniectomy, n (%)	4 (2.3)		0 (0.0)	0 (0.0)	2 (8.3)	1 (1.6)	1 (1.5)	0.41
Fraction of MCA territory								
infarcted, n (%)								0.49
< 1/3	68 (38.6)		5 (29.4)	1 (11.1)	7 (29.2)	24 (40.7)	31 (46.3)	
between 1/3 and 2/3	50 (28.4)		6 (35.3)	3 (33.3)	8 (33.3)	18 (30.5)	15 (22.4)	
> 2/3	58 (33.0)		6 (35.3)	5 (55.6)	9 (37.5)	17 (28.8)	21 (31.3)	
Stroke side								0.72
Left	94 (54.3)		8 (50.0)	6 (66.7)	14 (60.9)	28 (48.3)	38 (56.7)	
Right	79 (45.7)		8 (50.0)	3 (33.3)	9 (39.1)	30 (51.7)	29 (43.3)	
IV tPA, n (%)	76 (48.4)		8 (57.1)	6 (85.7)	12 (52.2)	23 (43.4)	27 (45.0)	0.26
Smoking, n (%)	91 (53.2)		6 (35.3)	1 (16.7)	12 (50.0)	31 (54.4)	41 (61.2)	0.12
Atrial fibrillation, n (%)	35 (19.7)		5 (29.4)	1 (11.1)	7 (29.2)	11 (18.0)	11 (16.4)	0.51
Diabetes mellitus, n (%)	32 (18.0)		4 (23.5)	1 (11.1)	7 (29.2)	11 (18.0)	9 (13.4)	0.45
Hypertension, n (%)	100 (56.2)		9 (52.9)	4 (44.4)	15 (62.5)	30 (49.2)	42 (62.7)	0.50
Hyperlipedemia, n (%)	51 (28.7)		4 (23.5)	2 (22.2)	6 (25.0)	22 (36.1)	17 (25.4)	0.64
Coronary artery disease, n (%)	51 (28.7)		4 (23.5)	2 (22.2)	6 (25.0)	22 (36.1)	17 (25.4)	0.64
*p<0.05 statistically significant								

Seventy-six (42.7%) of patients underwent IV tPA therapy prior to endovascular treatment (Table 2). Only four patients (2.2%) required a hemicraniectomy with no statistically significant association found between TICI score and hemicraniectomy (p=0.41) or between administration of IV tPA and hemicraniectomy (p=0.36). However, primary occlusion site was found to influence TICI score (p=0.045) but did not influence mortality (p=0.45) (Table 3).

**Table 2:** Baseline and selected characteristics of study patients by IV tPA administration IV tPA: intravenous tissue plasminogen activator; TICI: Thrombolysis in Cerebral Infarction score; MCA: middle cerebral artery.

Table 2. Baseline and selected characteristics of study patients by IV tPA administration					
Variable	Total N=178		no tPA n=81	tPA n=76	P-value
						Gender, n (%)					0.19
Male	87 (55.4)		49 (60.5)	38 (50.0)	
Female	70 (44.6)		32 (39.5)	38 (50.0)	
Race, n (%)					0.50
White	95 (64.2)		51 (65.4)	44 (62.9)	
Black	51 (34.5)		25 (32.0)	26 (37.1)	
Asian	2 (1.3)		2 (2.6)	0 (0.0)	
Hemicraniectomy, n (%)	4 (2.6)		1 (1.2)	3 (4.0)	0.36
TICI score, n (%)					0.26
grade 0	14 (8.9)		6 (7.4)	8 (10.5)	
grade 1	7 (4.5)		1 (1.2)	6 (7.9)	
grade 2a	23 (14.6)		11 (13.6)	12 (15.8)	
grade 2b	53 (33.8)		30 (37.1)	23 (30.3)	
grade 3	60 (38.2)		33 (40.7)	27 (35.5)	
Fraction of MCA territory					
infarcted, n (%)					0.85
< 1/3	60 (38.7)		32 (40.5)	28 (36.8)	
between 1/3 and 2/3	45 (29.0)		23 (29.1)	22 (29.0)	
> 2/3	50 (32.3)		24 (30.4)	26 (34.2)	
Stroke side					0.07
Left	81 (53.3)		46 (60.5)	35 (46.1)	
Right	71 (46.7)		30 (39.5)	41 (53.9)	
Smoking, n (%)	84 (56.0)		49 (62.8)	35 (48.6)	0.08
Atrial fibrillation, n (%)	31 (19.8)		17 (21.0)	14 (18.4)	0.69
Diabetes mellitus, n (%)	28 (17.8)		14 (17.3)	14 (18.4)	0.85
Hypertension, n (%)	87 (55.4)		39 (48.2)	48 (63.2)	0.06
Hyperlipedemia, n (%)	45 (28.7)		20 (24.7)	25 (32.9)	0.26
Coronary artery disease, n (%)	15 (9.6)		6 (7.4)	9 (11.8)	0.35
*p<0.05 statistically significant					

**Table 3:** Primary occlusion site of study patients by TICI scores TICI: Thrombolysis in Cerebral Infarction score; ACA: anterior cerebral artery; BA: brachial artery; CCA: common carotid artery; ICA: internal carotid artery.

Table 3. Primary occlusion site of study patients by TICI scores								
			TICI Scores					
Variable	Total N=178		Grade 0 n=17	Grade 1 n=8	Grade 2a n=24	Grade 2b n=61	Grade 3 n=67	P-value
								Primary occlusion site								0.045*
ACA	6 (3.4)		2 (11.8)	1 (11.1)	1 (4.2)	1 (1.6)	1 (1.5)	
BA	5 (2.8)		1 (5.9)	0 (0.0)	0 (0.0)	1 (1.6)	3 (4.5)	
CCA	12 (6.8)		0 (0.0)	0 (0.0)	4 (16.7)	4 (6.6)	4 (6.1)	
ICA	52 (29.4)		5 (29.4)	1 (11.1)	6 (25.0)	34 (39.3)	16 (24.2)	
M1	79 (44.6)		8 (47.1)	4 (44.4)	10 (41.7)	23 (37.7)	34 (51.5)	
M2	22 (12.4)		1 (5.9)	2 (22.3)	3 (12.5)	8 (13.1)	8 (12.1)	
M4	1 (0.6)		0 (0.0)	1 (11.1)	0 (0.0)	0 (0.0)	0 (0.0)	
*p<0.05 statistically significant								

Of the patients receiving endovascular treatment, 38.2% (n=60) achieved TICI3, 33.8% (n=53) achieved TICI2b, 14.6% (n=23) achieved TICI2a, and 13.4% (n=21) achieved a TICI score of 0 or 1. TICI score was found to influence mortality (p=0.02) (Table 4).

**Table 4:** Baseline and selected characteristics of study patients by vital status IV tPA: intravenous tissue plasminogen activator; TICI: Thrombolysis in Cerebral Infarction score; MCA: middle cerebral artery.

Table 4. Baseline and selected characteristics of study patients by vital status						
Variable	Total N=178		Living n=123	Deceased n=55	P-value	
						Gender, n (%)					0.51	
Male	97 (54.5)		65 (52.8)	32 (58.2)		
Female	81 (45.5)		58 (47.2)	23 (41.8)		
Race, n (%)					0.05	
White	110 (65.5)		73 (62.9)	37 (71.1)		
Black	56 (33.3)		43 (37.1)	13 (25.0)		
Asian	2 (1.2)		0 (0.0)	2 (3.9)		
Hemicraniectomy, n (%)	4 (2.3)		3 (2.4)	1 (1.8)	1.00	
IV tPA, n (%)	76 (48.4)		56 (52.8)	20 (39.2)	0.11	
TICI score, n (%)					0.02*	
grade 0	17 (9.5)		7 (5.7)	10 (18.2)		
grade 1	9 (5.1)		4 (3.3)	5 (9.1)		
grade 2a	24 (13.5)		16 (13.0)	24 (14.5)		
grade 2b	60 (33.7)		48 (39.0)	60 (21.8)		
grade 3	68 (38.2)		48 (39.0)	68 (36.4)		
Fraction of MCA territory						
infarcted, n (%)					<0.0001*	
< 1/3	69 (39.7)		62 (51.6)	7 (13.0)		
between 1/3 and 2/3	49 (28.2)		32 (26.7)	17 (31.5)		
> 2/3	56 (32.2)		26 (21.7)	30 (55.5)		
Stroke side					0.35	
Left	92 (54.4)		62 (52.1)	30 (60.0)		
Right	77 (45.6)		57 (47.9)	20 (40.0)		
Smoking, n (%)	91 (53.2)		65 (54.2)	26 (51.0)	0.70	
Atrial fibrillation, n (%)	35 (19.7)		31 (25.2)	4 (7.3)	0.005*	
Diabetes mellitus, n (%)	32 (18.0)		22 (17.9)	10 (18.2)	0.96	
Hypertension, n (%)	100 (56.2)		74 (60.2)	26 (47.3)	0.11	
Hyperlipedemia, n (%)	51 (28.7)		43 (35.0)	8 (14.6)	0.005*	
Coronary artery disease, n (%)	20 (11.2)		18 (14.6)	2 (3.6)	0.03*	
*p<0.05 statistically significant						

Additional logistic regression analysis demonstrated a statistically significant association between all three variables of TICI score, IV tPA use, and fraction of MCA vascular territory infarcted and death (Tables 5-6). TICI score had an odds ratio (OR) of 0.64 (95% CI 0.42-0.96, p = 0.033) when compared with death. IV tPA use had and OR of 0.37 (95% CI 0.17-0.83, p = 0.016) when compared with death. Lastly, fraction of MCA vascular territory infarcted had an OR of 3.34 (95% CI 2.04-5.54, p < 0.0001) when compared with death.

**Table 5:** Primary occlusion site of study patients by vital status BA: brachial artery; CCA: common carotid artery; ICA: internal carotid artery.

Table 5. Primary occlusion site of study patients by vital status					
Variable	Total N=178		Living n=123	Deceased n=55	P-value
					Primary occlusion site					0.45
A1	3 (1.7)		2 (1.7)	1 (1.8)	
A2	2 (1.1)		1 (0.8)	1 (1.8)	
A3	1 (0.6)		1 (0.8)	0 (0.0)	
BA	5 (2.8)		2 (1.7)	3 (5.5)	
CCA	12 (6.8)		9 (7.4)	3 (5.5)	
ICA	51 (29.0)		36 (29.7)	15 (27.2)	
M1	79 (44.9)		52 (43.0)	27 (49.1)	
M2	22 (12.5)		18 (14.9)	4 (7.3)	
M4	1 (0.6)		0 (0.0)	1 (1.8)	
*p<0.05 statistically significant					

**Table 6:** Logistic regression modeling death as outcome TICI: Thrombolysis in Cerebral Infarction score; MCA: middle cerebral artery; IV tPA: intravenous tissue plasminogen activator.

Table 6. Logistic regression modeling death as outcome			
Variable	Odds ratio	95% Confidence Interval	P-value
TICI	0.64	0.42 - 0.96	0.033*
IV tPA	0.37	0.17 - 0.83	0.016*
Fraction of MCA territory infarcted	3.34	2.04 - 5.54	<0.0001*
*p<0.05 statistically significant			

## Discussion

In 2013, a consensus statement defined the target endovascular recanalization after AIS as TICI2b, which is defined as the restoration of more than two-thirds of the total downstream territory of the blood vessel affected [22]. Recent studies have re-examined this definition of successful reperfusion, obtaining significantly better outcomes when obtaining a TICI3 score [9-11]. In a retrospective analysis of 222 patients, Dargazanli et al. found a significant decrease in intracranial hemorrhage rates as well as better functional outcomes when achieving TICI3 compared to TICI2b [10]. In a retrospective analysis of 262 patients, Kleine et al. also found a significant improvement in neurologic outcomes when obtaining TICI3 recanalization [11].

Mechanical thrombectomy devices have been shown to save large amounts of penumbra when achieving either TICI2b or TICI3 [23]. Sporns et al. showed a decrease in the frequency of hemicraniectomy following an occlusion of the MCA after the introduction of stent retrievers, illustrating the effectiveness of these devices [21]. These devices have also led to better clinical outcomes and higher rates of recanalization when compared with IV thrombolysis [6].

In malignant MCA infarctions, decompressive hemicraniectomy can be a life-saving procedure [14]. The current study sought to investigate the rate of hemicraniectomy after IAT in patients with acute MCA vascular territory infarctions. Of a total of 178 patients that met inclusion criteria, only four patients subsequently underwent decompressive hemicraniectomy regardless of the size of MCA vascular territory infarcted. The percentage of patients undergoing subsequent hemicraniectomy in this study, 2.2%, is lower than recent studies obtaining results of 8.2% and 8.7% [21,24]. These studies together suggest that despite patients presenting with large ischemic strokes, early IAT may reduce the need for hemicraniectomy. Furthermore, reducing the need for hemicraniectomy inherently reduces the rates of subsequent cranioplasty, a procedure with a complication rate reported as high as 36.6% [25]. Therefore, early IAT may reduce the need for two additional procedures and the possible morbidity associated with each.

Caution has to be applied when interpreting these results, however, given the limitations in statistical analysis inherent to this study secondary to the small number of hemicraniectomy patients. In the clinical setting, some patients are not considered candidates for hemicraniectomy due to other medical comorbidities making them poor surgical candidates or due to family and patient wishes for end of life care. The presence of these patients in our study population could artificially reduce our numbers of decompressive hemicraniectomy after AIS.

When comparing TICI score achieved to site of vascular occlusion, a statistically significant correlation was found with more proximal occlusions having higher rates of TICI2b and TICI 3 recanalization. This relationship makes clinical sense as proximal occlusions are more easily treated by endovascular intervention. Although TICI score was not found to be statistically correlated with hemicraniectomy (p = 0.41), logistic regression analysis did show correlation with mortality (p = 0.033) with OR 0.64. This suggests that as TICI score improves, rate of mortality decreases. Additionally, IV tPA use decreased mortality (OR 0.37, p = 0.016). As fraction of MCA vascular territory infarcted increased, patient mortality increased as well (OR 3.34, p < 0.0001). These findings support what is seen clinically in patients with MCA ischemic stroke.

## Conclusions

IAT may offer a protective advantage against the need for decompressive hemicraniectomy in patients, regardless of TICI reperfusion. The incidence of decompressive hemicraniectomy after AIS was not statistically correlated with TICI score, IV tPA use, or location of vascular occlusion although patient mortality was significantly associated with these same factors. Future case-control studies are needed to compare hemicraniectomy rates in patients that undergo IAT versus those who do not. Additional studies investigating outcomes when comparing variation in size of and timing for hemicraniectomy after IAT as well as cranioplasty are underway at our institution and may provide further guidance for standardization of practice.

## References

[1] Seshadri S and Wolf PA (2007). Lifetime risk of stroke and dementia: current concepts, and estimates from the Framingham Study. Lancet Neurol.

[2] Fischer U, Mono ML, Schroth G (2013). Endovascular therapy in 201 patients with acute symptomatic occlusion of the internal carotid artery. Eur J Neurol.

[3] Castano C, Dorado L, Guerrero C (2010). Mechanical thrombectomy with the Solitaire AB device in large artery occlusions of the anterior circulation: a pilot study. Stroke.

[4] Schwaiger BJ, Kober F, Gersing AS (2016). The pREset stent retriever for endovascular treatment of stroke caused by MCA occlusion: safety and clinical outcome. Clin Neuroradiol.

[5] Nogueira RG, Lutsep HL, Gupta R (2012). Trevo versus Merci retrievers for thrombectomy revascularisation of large vessel occlusions in acute ischaemic stroke (TREVO 2): a randomised trial. Lancet.

[6] Berkhemer OA, Fransen PS, Beumer D (2015). A randomized trial of intraarterial treatment for acute ischemic stroke. N Engl J Med.

[7] Marks MP, Lansberg MG, Mlynash M (2014). Correlation of AOL recanalization, TIMI reperfusion and TICI reperfusion with infarct growth and clinical outcome. J Neurointerv Surg.

[8] Higashida RT, Furlan AJ (2003). Trial design and reporting standards for intra-arterial cerebral thrombolysis for acute ischemic stroke. Stroke.

[9] Almekhlafi MA, Mishra S, Desai JA (2014). Not all “successful” angiographic reperfusion patients are an equal validation of a modified TICI scoring system. Interv Neuroradiol.

[10] Dargazanli C, Consoli A, Barral M (2017). Impact of modified TICI 3 versus modified TICI 2b reperfusion score to predict good outcome following endovascular therapy. AJNR Am J Neuroradiol.

[11] Kleine JF, Wunderlich S, Zimmer C, Kaesmacher J (2017). Time to redefine success? TICI 3 versus TICI 2b recanalization in middle cerebral artery occlusion treated with thrombectomy. J Neurointerv Surg.

[12] Chaudhry NS, Shah AH, Ferraro N, Snelling BM, Bregy A, Madhavan K, Komotar RJ (2013). Predictors of long-term survival in patients with glioblastoma multiforme: advancements from the last quarter century. Cancer Invest.

[13] Delashaw JB, Broaddus WC, Kassell NF (1990). Treatment of right hemispheric cerebral infarction by hemicraniectomy. Stroke.

[14] Juttler E, Schwab S, Schmiedek P (2007). Decompressive Surgery for the Treatment of Malignant Infarction of the Middle Cerebral Artery (DESTINY): a randomized, controlled trial. Stroke.

[15] Juttler E, Bosel J, Amiri H, Schiller P, Limprecht R, Hacke W, Unterberg A (2011). DESTINY II: Decompressive Surgery for the Treatment of Malignant Infarction of the Middle Cerebral Artery II. Int J Stroke.

[16] Hofmeijer J, Kappelle LJ, Algra A (2009). Surgical decompression for space-occupying cerebral infarction (the Hemicraniectomy After Middle Cerebral Artery infarction with Life-threatening Edema Trial [HAMLET]): a multicentre, open, randomised trial. Lancet Neurol.

[17] Vollman AT, Bruno CA, Dumeer S, Malone H, Meyers PM (2014). Angiographic warning of hemorrhagic transformation after stent retriever thrombectomy procedure. J Neurointerv Surg.

[18] Arkadir D, Eichel R, Cohen JE (2010). Decompressive hemicraniectomy improves outcome in patients with failed arterial recanalization after acute carotid artery occlusion. Neurol Res.

[19] Rahme R, Curry R, Kleindorfer D (2012). How often are patients with ischemic stroke eligible for decompressive hemicraniectomy?. Stroke.

[20] Adeoye O, Hornung R, Khatri P, Ringer A, Kleindorfer D (2011). The rate of hemicraniectomy for acute ischemic stroke is increasing in the United States. J Stroke Cerebrovasc Dis.

[21] Sporns PB, Minnerup J, Warneke N (2017). Impact of the implementation of thrombectomy with stent retrievers on the frequency of hemicraniectomy in patients with acute ischemic stroke. Clin Neuroradiol.

[22] Zaidat OO, Yoo AJ, Khatri P (2013). Recommendations on angiographic revascularization grading standards for acute ischemic stroke: a consensus statement. Stroke.

[23] Friedrich B, Kertels O, Bach D, Wunderlich S, Zimmer C, Prothmann S, Förschler A (2014). Fate of the penumbra after mechanical thrombectomy. AJNR Am J Neuroradiol.

[24] Suyama K, Horie N, Hayashi K, Nagata I (2014). Nationwide survey of decompressive hemicraniectomy for malignant middle cerebral artery infarction in Japan. World Neurosurg.

[25] Li A, Azad TD, Veeravagu A, Bhatti I, Long C, Ratliff JK, Li G (2017). Cranioplasty complications and costs: a national population-level analysis using the marketscan longitudinal database. World Neurosurg.

